# Domestic elder abuse and associated factors in elderly women in Tehran, Iran

**DOI:** 10.4178/epih.e2018055

**Published:** 2018-11-10

**Authors:** Negar Piri, Parisa Taheri Tanjani, Soheila Khodkarim, Koorosh Etemad

**Affiliations:** 1 School of Public Health and Safety, Shahid Beheshti University of Medical Sciences, Tehran, Iran; 2 Department of Internal Medicine, School of Medicine, Shahid Beheshti University of Medical Sciences, Tehran, Iran; 3 Department of Biostatistics, School of Public Health and Safety, Shahid Beheshti University of Medical Sciences, Tehran, Iran; 4 Department of Epidemiology, School of Public Health and Safety, Environmental and Occupational Hazards Control Research Center, Shahid Beheshti University of Medical Sciences, Tehran, Iran

**Keywords:** Domestic, Elder abuse, Aged, Women, Risk factors, Iran

## Abstract

**OBJECTIVES:**

Elderly people are often abused in many ways, with serious and lasting consequences. Elder abuse remains one of the most hidden forms of family conflict, and its frequency is anticipated to be rising in many countries that are rapidly experiencing population aging. The purpose of this study was to investigate the status of domestic elder abuse in elderly women in Tehran, Iran and to identify associated factors.

**METHODS:**

This cross-sectional study was conducted in 2017 among 260 women aged 60 and over, selected through multistage random sampling. Information on abuse and its risk factors was collected through interviews with the elderly in their homes. In order to measure domestic elder abuse, the validated Domestic Elderly Abuse Questionnaire was used. The ordinal logistic test was used in Stata version 12 to identify factors related to elder abuse.

**RESULTS:**

Overall, 90.4% of the subjects had experienced at least 1 type of abuse, among which authority deprivation (68.5%) was the most common and rejection (11.2%) the least common. The ordinal logistic analysis showed that the likelihood of experiencing more severe severe abuse was significantly higher in elderly people over 72 than in those aged 60-62 years (odds ratio, 2.07; 95% confidence interval, 1.03 to 4.17).

**CONCLUSIONS:**

Domestic elder abuse was found to be common in older women. Old age was an important risk factor for domestic elder abuse in elderly women in Tehran. Managing and preventing elder abuse is an important issue that needs to be addressed.

## INTRODUCTION

Old age is one of the most vital periods of human life [1]. According to the most recent World Health Organization (WHO) report, the number of people over 60 is expected to double by 2050, requiring fundamental social changes [2]. In Iran, the Population and Housing Census report in 2015 estimated the number of people over 60 as 7,414,091, forming 9.3% of the overall population [3].

The consequences of an increasing number of elderly people in the family include negative effects on their physical and mental status, economic pressures, mental disorders and emotional tensions, lack of responsibility and tolerance, and individual fatigue and social isolation of family members, and these consequences can be followed by the emergence of anti-social behaviors and increasing violence [4]. Under such circumstances, families are often not prepared to take care of the elderly, and in combination with other social factors such as urbanization, modernity, changing traditional values, and the contrast between the value systems of the new and old generations, families sometimes do not play their proper role for the elderly, who may be exposed to domestic elder abuse [5]. A common definition of elder abuse approved by WHO and the International Network for the Prevention of Elder Abuse is as follows: “Elder abuse is a single or repeated act, or lack of appropriate action, occurring within any relationship where there is an expectation of trust, which causes harm or distress to an older person.” [6,7].

The WHO reported that in 2017, around 1 out of 6 old people experienced some form of abuse [8], and the results of a meta-analysis in 2017 indicated that in the last year, 15.7% of people aged 60 years and over underwent some form of abuse [9].

Abuse often occurs in family settings, such as at home [10,11], and the first perpetrators of elder abuse are the family members of elderly individuals [10]. Elder abuse is a multidimensional problem [11]; some explanations have stressed the victim’s features and/or those of the abusers, while others have emphasized living conditions or family status [12]. Most studies have reported that women, physical disability and functional impairment, dependence on others, poor physical and/or mental health, low income or poverty, and lack of social support were major risk factors associated with elder abuse [6,10,12-14].

Abuse exerts negative effects on mental and physical capacity, social status, and structures [15], and is associated with negative outcomes, including mental distress, morbidity, and death [16]. Furthermore, this issue is very costly in terms of the possibility of transfer to a nursing home and hospitalization [7].

Gender is an important factor in aging, and women are better represented than men in higher age groups. This issue was vividly illustrated by the 2016 Iran-wide census, in which more than 50% of the elderly were women [3]. Furthermore, the high prevalence of age-related diseases in older women [17], and the failure to identify many cases of abuse, especially in women, make abuse a serious threat and harm to the elderly; thus, identifying the prevalence of elder abuse and the factors associated with its occurrence could be a major step in determining how to prevent abuse and in facilitating protection of the elderly. For these reasons, the present study aimed to present up-to-date data on the abuse of elderly women and related factors in Tehran, Iran.

## MATERIALS AND METHODS

This was a cross-sectional, descriptive-analytical study conducted in the urban area of Tehran, the capital of Iran, among elderly women who were covered by health centers associated with Shahid Beheshti University of Medical Science, which is one of the biggest universities of Iran. Its health centers include many areas in Tehran, with a population of about 4,122,406, of whom 278,721 are old women. The aging population in these areas is increasing rapidly, which has drawn increasing attention to the problems faced by this age group.

### Subjects and sampling

The study population consisted of elderly women, aged 60 years and over, covered by Shahid Beheshti University of Medical Science health centers in Tehran. Sampling was carried out using a multistage random sampling method. First, the health centers included in each of the 3 healthcare networks being covered by Shahid Beheshti University of Medical Science in Tehran (the east healthcare network, the north healthcare network, and district 1 of the Shemiranat healthcare network) were defined based on the locations of the 22 districts (districts 1, 3, 4, 7, 8, 12, 13, 14, and 15) of Tehran. Then in each district, a health center was randomly selected (in district 1, two centers were chosen), and finally, based on the list of elderly women treated at each selected health center, 260 elderly women (proportional to the number of elderly women at each of the health centers) were systematically selected.

### Data collection method

Data were collected by interviewing the elderly in their homes from July to October 2017. The interviews were conducted using a structured interview protocol by the researcher and providers of the health centers. Phone calls were made to reach an initial agreement concerning the home interview with each of the selected individuals; then, after explaining the purpose of the study and obtaining informed consent during the visit, participants were interviewed privately.

### Data collection tool

The interview protocol included 4 sections: (1) cognitive status assessment; (2) general and demographic characteristics; (3) family characteristics; and (4) domestic elder abuse assessment.

The items included in the interview were scored on numerical scales, using predefined categories.

#### Cognitive status

For cognitive screening of the elderly, the 3-word test from the Mini–Mental State Examination (MMSE) instrument was used. If the person was eligible to enter the study in terms of cognitive status, they proceeded with the interview, but those who were not eligible were substituted with a person higher or lower on the list of elderly people maintained by the health centers. The 30-item MMSE to rate cognitive levels was developed by Folstein et al. [18], and the Persian version was validated by Foroughan et al. [19]. This tool has satisfactory reliability and validity (Cronbach alpha, 0.78). The 3-word test consists of 2 parts: memorizing and recalling a set of 3 words. A score of 0 for memorizing or a score of fewer than 3 points for recalling indicates a cognitive problem.

#### General and demographic characteristics

The characteristics reviewed in this section included: age, education level, marital status, number of children, living status, employment status, economic dependency, insurance status, dependence on supporting organizations, smoking, hookah use, drug use, and the presence chronic diseases (hypertension, cardiovascular disease and diabetes).

#### Family characteristics

The following variables were examined to evaluate family characteristics: monthly household income, housing type, house ownership type, and household size.

#### Domestic elder abuse assessment

Data on elder abuse were collected using the Domestic Elderly Abuse Questionnaire, which was developed and validated by Heravi-Karimooi et al. [20] and is an appropriate tool for investigating elder abuse in Iranian families that can be used in different situations. The Domestic Elderly Abuse Questionnaire contains 49 items with 8 subscales of emotional neglect, care neglect, financial neglect, authority deprivation, psychological abuse, physical abuse, financial abuse, and rejection. The tool items include the options “yes,” “no,” and “not applicable”. The option “not applicable” indicates that the items are not consistent with the life of the respondent. The obtained scores range from 0 to 100, and higher scores indicate more severe abuse symptoms. This tool was validated based on its face validity and content validity (CVI, 0.92), construct validity, high internal consistency (Cronbach alpha, 0.90 to 0.97), and reliability through re-testing (0.99) [20].

### Inclusion criteria

The inclusion criteria included the provision of informed consent, age 60 and older, women, psychological health based on the 3-word cognitive test, and the lack of severe cognitive impairment (dementia, Alzheimer’s disease and etc.).

### Data analysis

In order to analyze the data, Stata version 12 (StataCorp., College Station, TX, USA) was used. To explain the relationship between the variables analyzed in this study and levels of domestic elder abuse, ordinal logistic regression (proportional odds model) was used. To fit the proportional odds model, the total score of elder abuse was divided into 3 levels: minimal (scores 0-10), moderate (scores 10-30), and severe (scores higher than 30).

To fit the multiple ordinal logistic regression model, variables with a significance level lower than 0.2 in the simple ordinal logistic regression model (age, education level, family size, and income) were included in the model. The p<0.05 was taken as the statistical significance level.

### Ethics statements

Ethical approval was granted by the Shahid Beheshti University of Medical Sciences Research Ethics Committee (Ir.sbmu.PHNS.REC.1396.13).

## RESULTS

Table 1 presents the study subjects’ general characteristics, divided into their demographic and general characteristics and their family characteristics. This table also presents the number and percentage of participants who experienced abuse stratified according to the variables studied. The mean age of the study subjects was 67.63±6.62 years, and the age group of 60-62 years made up the largest proportion (27.3%). More than half of the elderly (53.5%) were literate, 161 (61.9%) were married, and 177 (68.1%) suffered from a chronic disease; other data are shown in Table 1.

In Table 2, the mean total score for elder abuse and the mean scores of the 8 subscales are given. The mean score of abuse among the study subjects was 19.29±19.03, and more than 90% of the subjects (n=235) had experienced at least 1 type of abuse. Authority deprivation (68.5%) and psychological abuse (63.5%) were the most common types of abuse, and rejection (11.2%) was the least common type.

The ordinal logistic regression analysis indicated that the likelihood of experiencing more severe abuse in illiterate elderly women was almost 1.20 times greater than in literate women, and in the elderly with a household size ≥2, the odds of abuse were 1.48 times higher than in the elderly with a smaller household. Moreover, the odds of experiencing more severe abuse in the elderly with an income lower than 8 million rials were 1.11 times higher than in the elderly with an income above 15 million rials. Furthermore, the odds of experiencing more severe abuse in those older than 72 years were 2.07 times greater than those of the elderly who were 60-62 years of age. However, only age showed a significant association with abuse (p=0.04). The variables of employment status, insurance status, dependence on supporting organizations, smoking, hookah use, and drug use were removed from the ordinal logistic analysis due to the small number of samples at some levels. The results of the univariate and multivariate ordinal logistic regression analysis are shown in Table 3.

## DISCUSSION

The objective of the present study was to evaluate the status of abuse and some of its associated factors among elderly women living in Tehran. The mean total abuse score was 19.29±19.03, and 90.4% of respondents reported having experienced some form of abuse, of which authority deprivation (68.5%) and psychological abuse (63.5%) were the most common forms and rejection (11.2%) was the least common. These results are consistent with those of some previous studies, but very different from those of others.

In a meta-analysis of 50 studies from throughout the world, the overall prevalence of abuse of elderly women was estimated to be 14.1%, and estimates were obtained for the prevalence of psychological abuse (11.8%), neglect (4.1%), financial abuse (3.8%), sexual abuse (2.2%), and physical abuse (1.9%) [21]. In a study in the city of Pune, India, abuse was reported among 47.0% of elderly women [22]. In the Prevalence Study of Abuse and Violence against Older Women (AVOW) study, 28.1% of old women underwent abuse; as such, those results are dramatically different from those of the present study. In the AVOW study, psychological abuse and physical violence were the most and least common forms of abuse, respectively [17].

Turning to studies conducted in Iran, in the study by Khalili et al. [23], similar to the present study, many elderly women (80.0%) stated that they had experienced abuse, but unlike the current study, financial abuse was the most common type of abuse. In studies conducted by Alizadeh-Khoei et al. [24], and Hosseini et al. [25], abuse was only reported among 14.7 and 17.4% of the elderly, and physical abuse was the most common type in both studies.

One of the reasons for the differences in the reported prevalence is that there is no obvious and unique definition of elder abuse, meaning that the definition and estimation of elder abuse vary across environments and studies. Other reasons include the lack of unique standards and tools, variation in data collection methods, and the existence of various values and norms in different societies with resulting differences in definitions and perceptions of acceptable behavior. Nonetheless, some likely reasons for the high prevalence of elder abuse in Iranian families include rapid changes in family values and structures, which have caused the elderly, who always strive to preserve their beliefs, traditions, and values, to experience conflict with the existing circumstances and their families, resulting in conflicts among Iranian elderly and their families. Furthermore, elderly Iranian women by tradition occasionally undertake the responsibility of caring for their children and grandchildren against their will, and this phenomenon causes conflicts and opposition in Iranian families; in addition, restrictions on working outdoors may result in the elderly becoming economically dependent on their family members, which is a common background for abuse and financial neglect.

In the present research, only old age was identified as a significant risk factor for elder abuse. Most studies, such as those conducted in the US [26,27], Malaysia [28], Europe (the AVOW study) [29], and a study by Ghodoosi et al. [4], in contrast with the present research, reported that the elderly in younger age groups were exposed to more abuse. In contrast, some other studies in Mexico [30], Portugal [31], and Iran [32] reported findings similar to those of this study, with a higher prevalence of abuse among the elderly in older age groups. More widespread abuse among older people can be explained through the possibility that diseases related to age and the vulnerability of old individuals provide the circumstances for elder abuse. As a consequence, older women are exposed to a greater risk of abuse and its outcomes; this is compounded by the fact that relatively light forms of physical abuse, such as pushing, can lead to serious injuries in the elderly [9].

In the present study, the results also indicated that elderly women with a lower income were more frequently exposed to abuse than those with a higher income. These findings are consistent with studies conducted in Mexico, Ireland, India, and the US [33]. In a study by Nori et al. [34], a statistically significant difference in elder abuse was found between those with a monthly income lower than 0.5 million rials and those with a monthly income over 5 million rials.

In a study by Skirbekk & James [35], it was found that only having few or no years of formal education was associated with abuse. Although the current study found that illiterate participants were exposed to higher odds of abuse, that relationship was not significant. Other studies [32,36,37], congruent with the current study, did not show a significant relationship between higher abuse and lower education. In contrast, the study by Alizadeh-Khoei et al. [24] suggested lower odds of abuse and neglect in the elderly with primary or higher education compared to their illiterate counterparts. The views of elderly women, including an emphasis on their traditional gender role and valuing the domestic role, cause them to be less inclined to pursue education and employment; this makes them more dependent on their family and relatives, and increases their likelihood of experiencing abuse [38].

In this study, similar to the AVOW study, the odds of abuse were higher among the elderly with a larger household size [17]. In contrast, in the study by Skirbekk & James [35], the likelihood of abuse in larger households was lower.

The results of this study and many other studies indicate that elderly women are exposed to many types of abuse. This is a very important health and social issue that requires attention and action, such as proper contextualization of healthy aging; informing the community and families about the physical, mental, emotional, and other needs of the elderly; and efforts by the government to provide support for the elderly to be more independent and better-educated as a way to promote their health. Furthermore, old age is an important risk factor for domestic elder abuse in women, and this is an important issue that needs to be addressed.

Since the elderly women in this study were only drawn from 3 urban districts of Tehran, including the north, east, and Shemiranat districts, and were also covered by health centers, it is possible that the characteristics of these participants differ from those of elderly women living in other districts of Tehran and those not covered by the health centers. Accordingly, the present study may not represent all dwellers of Tehran or all Iranian elderly people.

Furthermore, characteristics of the abusers and certain other important factors that could potentially influence elder abuse, such as socioeconomic status and other social factors, were not investigated in the present study.

## Figures and Tables

**Table 1.** General characteristics of the subjects studied

Characteristics			Total	Abused	Non-abused
Elderly	Age (yr)	60-62	71 (27.3)	61 (85.9)	10 (41.1)
		63-66	63 (24.2)	59 (93.7)	4 (6.3)
		67-72	69 (26.5)	62 (89.9)	7 (10.1)
		>72	57 (21.9)	53 (93.0)	4 (7.0)
	Education level	Illiterate	121 (46.5)	111 (91.7)	10 (8.3)
		Literate	139 (53.5)	124 (89.2)	15 (10.8)
	Marital status	Married	161 (61.9)	146 (90.7)	15 (9.3)
		Single	99 (38.1)	89 (89.9)	10 (10.1)
	No. of children	<4	96 (36.9)	86 (89.6)	10 (10.4)
		≥4	164 (63.1)	149 (90.9)	15 (9.1)
	Living status	Alone	48 (18.5)	42 (87.5)	6 (12.5)
		With spouse	76 (29.2)	69 (90.8)	7 (9.2)
		With children	50 (19.2)	46 (92.0)	4 (8.0)
		With spouse and children	86 (33.1)	78 (90.7)	8 (9.3)
	Employment status	Employed	2 (0.8)	2 (100.0)	N/A
		Housewife	238 (91.5)	216 (90.8)	22 (9.2)
		Retired	20 (7.7)	17 (85.0)	3 (15.0)
	Economic dependency	Yes	79 (30.4)	72 (91.1)	7 (8.9)
		No	181 (69.6)	163 (90.1)	18 (9.9)
	Insurance status	Yes	236 (90.8)	212 (89.8)	24 (10.2)
		No	24 (9.2)	23 (95.8)	1 (4.2)
	Dependence on supporting organizations	Yes	24 (9.2)	23 (95.8)	1 (4.2)
		No	236 (90.8)	212 (89.8)	24 (10.2)
	Smoking	Yes	6 (2.3)	6 (100.0)	N/A
		No	254 (97.7)	229 (90.2)	25 (9.8)
	Hookah use	Yes	15 (5.8)	15 (100.0)	N/A
		No	245 (94.2)	220 (89.8)	25 (10.2)
	Drug use	Yes	1 (0.4)	1 (100.0)	N/A
		No	259 (99.6)	234 (90.3)	25 (9.7)
	Chronic disease	Yes	177 (68.1)	162 (91.5)	15 (8.5)
		No	83 (31.9)	73 (88.0)	10 (12.0)
Family	Monthly household income (million rials)	<8	75 (28.8)	71 (94.7)	4 (5.3)
		8-10	57 (21.9)	52 (91.2)	5 (8.8)
		10-15	78 (30.0)	70 (89.7)	8 (10.3)
		>15	50 (19.2)	42 (84.0)	8 (16.0)
	Housing type	Apartment	147 (56.5)	132 (89.8)	15 (10.2)
		Detached house	113 (43.5)	103 (91.2)	10 (8.8)
	House ownership type	Owner	192 (73.8)	175 (91.1)	17 (8.9)
		Tenant	68 (26.2)	60 (88.2)	8 (11.8)
	Household size	≤2	152 (58.5)	137 (90.1)	15 (9.9)
		>2	108 (41.5)	98 (90.7)	10 (9.3)

Values are presented as number (%). N/A, not applicable.

**Table 2.** Mean score and prevalence of elder abuse and its subscales

	Yes	No	Mean±SD
Emotional neglect	117 (45.0)	143 (55.0)	41.34±47.44
Care neglect	100 (38.5)	160 (61.5)	18.54±29.42
Financial neglect	87 (33.5)	173 (66.5)	23.27±37.31
Authority deprivation	178 (68.5)	82 (31.5)	17.08±18.56
Psychological abuse	165 (63.5)	95 (36.5)	26.77±29.97
Physical abuse	36 (13.8)	224 (86.2)	6.73±19.09
Financial abuse	92 (35.4)	168 (64.6)	13.42±22.13
Rejection	29 (11.2)	231 (88.8)	7.43±23.02
Abuse (total)	235 (90.4)	25 (9.6)	19.29±19.03

Values are presented as number (%). SD, standard deviation.

**Table 3.** The ordinal logistic regression analysis of factors related to elder abuse

Characteristics		Univariate model			Multivariate model		
		*β* (SE)	Wald (p-value)	OR (95%CI)	*β* (SE)	Wald (p-value)	OR (95%CI)
Age (yr)	60-62	-	-	1.00 (reference)	-	-	1.00 (reference)
	63-66	0.19 (0.32)	0.57 (0.56)	1.20 (0.63, 2.29)	0.17 (0.34)	0.50 (0.61)	1.18 (0.60, 2.30)
	67-72	0.48 (0.32)	1.50 (0.13)	1.61 (0.86, 3.02)	0.51 (0.33)	1.55 (0.12)	1.67 (0.87, 3.20)
	>72	0.76 (0.32)	2.32 (0.02)	2.13 (1.12, 4.05)	0.73 (0.35)	2.05 (0.04)	2.07 (1.03, 4.17)
Education level	Literate	-	-	1.00 (reference)	-	-	1.00 (reference)
	Illiterate	0.40 (0.23)	1.74 (0.08)	1.49 (0.95, 2.35)	0.18 (0.26)	0.70 (0.48)	1.20 (0.71, 2.00)
Marital status	Single	-	-	1.00 (reference)	-	-	-
	Married	-0.13 (0.23)	-0.56 (0.57)	0.87 (0.54, 1.39)	-	-	-
No. of children	<4	-	-	1.00 (reference)	-	-	-
	≥4	0.12 (0.24)	0.50 (0.62)	1.12 (0.69, 1.81)	-	-	-
Living status	Alone	-	-	1.00 (reference)	-	-	-
	With spouse	-0.25 (0.34)	-0.73 (0.46)	0.77 (0.39, 1.51)	-	-	-
	With children	-13.60 (0.37)	-0.00 (1.00)	1.00 (047, 2.09)	-	-	-
	With spouse and children	0.18 (0.33)	0.55 (0.58)	1.19 (0.62, 2.31)	-	-	-
Economic dependency	No	-	-	1.00 (reference)	-	-	-
	Yes	0.11 (0.25)	0.45 (0.65)	1.12 (0.68, 1.83)	-	-	-
Chronic disease	Yes	-	-	1.00 (reference)	-	-	-
	No	0.13 (0.24)	0.54 (0.58)	1.14 (0.70, 1.84)	-	-	-
Monthly house-hold income (million rials)	>15	-	-	1.00 (reference)	-	-	1.00 (reference)
	10-15	0.02 (0.33)	0.05 (0.96)	1.01 (0.52, 1.96)	-0.10 (0.35)	-0.30 (0.76)	0.90 (0.45, 1.79)
	8-10	0.67 (0.36)	1.85 (0.06)	1.95 (0.96, 3.96)	0.55 (0.37)	1.45 (0.14)	1.72 (0.72, 3.61)
	<8	0.41 (0.33)	1.21 (0.22)	1.50 (0.77, 2.89)	0.11 (0.37)	0.29 (0.77)	1.11 (0.53, 2.31)
Housing type	Detached housing	-	-	1.00 (reference)	-	-	-
	Apartment	0.02 (0.23)	0.07 (0.94)	1.01 (0.64, 1.60)	-	-	-
House ownership type	Tenant	-	-	1.00 (reference)	-	-	-
	Owner	0.26 (0.26)	0.98 (0.32)	1.29 (0.77, 2.18)	-	-	-
Household size	≤2	-	-	1.00 (reference)	-	-	1.00 (reference)
	>2	0.29 (0.23)	1.27 (0.19)	1.34 (0.85, 2.12)	0.393 (0.24)	1.63 (0.10)	1.48 (0.92, 2.37)

SE, standard error; OR, odds ratio; CI, confidence interval.
